# Coaching unified sports: associations between perceived athlete improvement, barriers, and coach attitudes across five European countries

**DOI:** 10.3389/fpsyg.2025.1632589

**Published:** 2025-08-04

**Authors:** Maciej Wilski, Piotr Urbański, Roxana Ossian, Gabriela Papp Eniko, Nina Bracanovic Milovic, Ivan Radovic, Veronica Sedlackova, Eva Gazova, Nadira Sabanovic, Kada Delic Selimovic, Velibor Srdic, Srboljub Vukovic, Damir Ahmic, Vaiva Abramaviciute, Daiva Dabriliene, Ausra Kriskovieciene, Anna Nadolska

**Affiliations:** ^1^Department of Adapted Physical Activity, Poznan University of Physical Education, Poznan, Poland; ^2^Special Olympics Romania, Bucharest, Romania; ^3^School Center for Inclusive Education, Târgu Mureș, Romania; ^4^Special Olympics Montenegro, Podgorica, Montenegro; ^5^Special Olympics Slovakia, Bratislava, Slovakia; ^6^Special Olympics Bosnia and Herzegovina, Sarajevo, Bosnia and Herzegovina; ^7^Department of Sport Sciences, Panevropski Univerzitet Apeiron, Banja Luka, Bosnia and Herzegovina; ^8^Department of Education, Univerzitet u Travniku, Travnik, Bosnia and Herzegovina; ^9^Special Olympics Lithuania, Vilnius, Lithuania

**Keywords:** unified sports, inclusive coaching, developmental disabilities, perceived barriers, attitudes toward inclusion

## Abstract

**Purpose:**

This study aimed to examine how perceived athlete improvement, perceived barriers to implementation, and selected coaching characteristics are associated with coaches’ attitudes toward Unified Sports programs. The investigation focused on understanding the psychological and contextual factors that influence inclusive coaching engagement within a multi-country sample, rather than comparing national differences directly.

**Methods:**

A cross-sectional survey was conducted with 102 coaches involved in Unified Sports programs in five European countries. Participants completed a standardized questionnaire assessing their attitudes toward inclusion, perceived improvement in athletes with developmental disabilities and their non-disabled partners, perceived implementation barriers, and personal coaching background. Hierarchical multiple regression analysis was used to identify predictors of coaching attitudes.

**Results:**

Perceived improvement in athletes with developmental disabilities was the strongest and most consistent predictor of positive coaching attitudes. Coaches who reported greater perceived progress in these athletes were more likely to endorse inclusive beliefs. In contrast, perceived improvement in non-disabled partners, although generally rated highly, did not significantly predict coaching attitudes. Interestingly, coaches with prior experience working in disability sports and those with familial relationships to participating athletes expressed more skeptical views, suggesting that emotional involvement or cumulative exposure may introduce attitudinal strain. Although institutional, social, and logistical barriers to Unified Sports were widely recognized by participants, these factors did not independently predict attitudes once other variables were controlled for.

**Conclusion:**

The findings underscore the motivational role of observed progress among athletes with disabilities in shaping coach engagement and suggest that experiential factors such as previous involvement and personal ties may carry unanticipated emotional or structural challenges. These insights point to the importance of designing coach education and support programs that not only promote technical competence but also address emotional resilience and contextual demands. Strengthening these components may enhance the sustainability and effectiveness of Unified Sports initiatives worldwide.

## 1 Introduction

### 1.1 Unified sports and inclusive coaching context

Unified Sports, an initiative under the Special Olympics movement, is designed to promote social inclusion by integrating athletes with developmental disabilities (DD) and their peers without disabilities into joint sports teams. The program fosters mutual understanding, skill development, and social cohesion (McConkey et al., 2013). Research has demonstrated that participation in Unified Sports can enhance self-concept, social skills, and physical competence among athletes with DD (Bota et al., 2014; Pan and Davis, 2019). Furthermore, it has been shown that positive attitudes and support from coaches are critical to the success of these programs, as they play a key role in creating an inclusive team environment and facilitating meaningful interactions among athletes (Hassan and Lynch, 2014; Hammond et al., 2014; Svanelöv et al., 2020).

Despite the proven benefits of Unified Sports, various barriers limit the effectiveness and sustainability of these initiatives across different national and cultural contexts. One key challenge is the perception and attitudes of coaches, who play a pivotal role in shaping the experiences of athletes in inclusive sports settings. Their level of preparedness, personal beliefs, and the institutional structures supporting or hindering Unified Sports greatly influence participation and success rates (McConkey et al., 2021; Hassan et al., 2012). Wilski et al. (2012) found that participation in Unified Sports contributes to athletes’ personal development in three key areas: physical, mental, and social. Participants reported improvements in fitness, technical skills, and teamwork, emphasizing the importance of collaboration and trust among teammates. Additionally, the study highlighted that athletes experienced increased confidence, self-esteem, and enhanced communication skills, which facilitated more positive social interactions. A strong sense of belonging and friendships within the team environment played a vital role in shaping these outcomes, reinforcing the importance of structured and inclusive team engagement in fostering these benefits. These factors are closely linked to the coach’s attitude and role as a leader, as they are responsible for creating an environment that nurtures relationships, supports development, and strengthens team cohesion (Hassan et al., 2012; Dowling, 2014; Hammond et al., 2014).

### 1.2 Challenges and barriers in implementing unified sports

The attitudes and behaviors of coaches toward Unified Sports directly impact athlete participation, team dynamics, and the overall success of inclusion initiatives (McConkey et al., 2013; McConkey et al., 2021; Hassan et al., 2012; Svanelöv et al., 2020). Coaches play a pivotal role in fostering inclusive environments, and previous research suggests that their personal beliefs, level of preparedness, and external support systems significantly influence their engagement in Unified Sports (Rizzo, 1984; Conatser et al., 2002; Hammond et al., 2014; Dowling, 2014; McConkey et al., 2021). However, studies have also highlighted various barriers—organizational, psychological, and social—that shape coaches’ attitudes and willingness to engage in Unified Sports (Dowling, 2014).

One frequently cited challenge is the lack of formal training on how to coach athletes with DD, which has been consistently reported as a major barrier (Temple and Walkley, 1999; Hammond et al., 2014). Additionally, limited institutional and administrative support (Tsai and Fung, 2009; Grandisson et al., 2021), stereotypical beliefs and biases (Hammond et al., 2014; Hammond et al., 2019; Kandianos et al., 2023), and structural constraints such as access to facilities and resources (Khetani et al., 2015) further hinder the implementation of inclusive sports programs.

### 1.3 Factors shaping coaching attitudes

While coaching experience in inclusive settings is assumed to foster more positive attitudes toward Unified Sports, empirical evidence remains mixed. Some studies suggest that familiarity with athletes with DD may lead to increased confidence and a more inclusive coaching approach (Vargas et al., 2012; Hassan and Lynch, 2014; Mauro et al., 2021), yet the extent to which experience translates into positive attitudes depends on various contextual factors, including perceived challenges and the observable benefits of participation.

One key factor shaping coaching attitudes is the perception of athlete and partner improvements. While Unified Sports is intended to facilitate personal and athletic development for individuals with DD, the degree to which coaches perceive tangible improvements among athletes may influence their level of engagement and overall attitude toward the program. Some studies suggest that coaches derive motivation from observing athlete progress (Mageau and Vallerand, 2003; Moen and Federici, 2013; Sakalidis et al., 2023), yet it remains unclear whether this effect translates into sustained commitment to inclusive coaching environments. Conversely, if progress is not evident, coaches may question the effectiveness of Unified Sports, potentially influencing their attitudes in a more neutral or negative direction.

Another key factor frequently assumed to influence coaching attitudes is perceived barriers to participation. Coaches may face a range of obstacles, including organizational, financial, social, and logistical challenges, which may hinder their willingness to engage in Unified Sports. While some research suggests that these perceived barriers negatively affect coaching attitudes (Jaarsma et al., 2014; Ballas et al., 2022), not all coaches respond to these obstacles in the same way. Some may remain committed to inclusion efforts despite encountering difficulties, highlighting the complexity of how barriers influence coaching attitudes.

Beyond perceived barriers and improvements, coaching attitudes may also be shaped by additional contextual and demographic factors. While much of the research on Unified Sports has focused on the role of training and institutional support, fewer studies have examined how individual coach characteristics—such as their professional background, prior experience, or socio-demographic attributes—may interact with perceptions of barriers and athlete progress to shape attitudes toward Unified Sports. Understanding these additional factors is crucial in developing more targeted strategies to enhance coaching engagement in inclusive sports settings (Rizzo et al., 1997; Conatser et al., 2002; Hammond et al., 2014; MacDonald et al., 2016; Hammond, 2022; Orbán-Sebestyén et al., 2023).

### 1.4 Study objectives and research questions

This study aims to investigate how perceived barriers, perceptions of athlete improvement, and additional coaching characteristics are related to attitudes toward Unified Sports. Specifically, it addresses the following research questions:

1. How are perceptions of barriers and athlete progress associated with coaching attitudes toward Unified Sports?
2. Is there a relationship between prior experience in working with athletes with developmental disabilities and coaching attitudes?
3. Which additional coach-related characteristics are linked to attitudes toward Unified Sports?

By exploring these relationships, this study contributes to a deeper understanding of how coaching attitudes in Unified Sports are shaped by a combination of barrier perceptions, perceived athlete progress, and broader coach-related factors. The findings will provide empirical insights for training programs, policy development, and strategies aimed at enhancing the sustainability of Unified Sports across different national contexts.

## 2 Materials and methods

### 2.1 Participants

The target group for this study comprised coaches actively involved in current Unified Sports programs, with a key inclusion criterion requiring that all participants be currently coaching a Unified Sports team. This ensured that responses reflected firsthand experiences in an inclusive coaching environment. Unified Sports teams included both athletes with developmental disabilities and their peers without disabilities (partners), engaging in football or basketball training and competitions. These teams followed the Unified Sports model, which promotes equal participation, cooperative team dynamics, and mutual skill development among players of varying abilities.

A total of 172 questionnaires were collected across the five participating countries. However, to ensure data integrity, incomplete questionnaires were excluded from analysis, resulting in a final sample of 102 coaches with complete and valid responses. The selection of countries was based on their participation in a larger international initiative funded by the EEA and Norway Grants, which focused on promoting social inclusion through Unified Sports. These countries were involved in a coordinated implementation framework led by the Poznan University of Physical Education and had established partnerships with local Special Olympics organizations, allowing for consistent data collection procedures and shared methodological standards. Their inclusion ensured practical feasibility, existing program infrastructure, and organizational readiness to support the research activities. The sample included participants from Slovakia, Romania, Bosnia & Herzegovina, Montenegro, and Lithuania. The number of respondents per country is presented in Table 1, reflecting variation in national representation that limits between-country comparisons. All participants were involved in a broader initiative focused on fostering inclusion through sports for children with developmental disabilities, coordinated by Poznan University of Physical Education and funded by the EEA and Norway Grants Fund for Regional Cooperation.

**Table 1:** Descriptive characteristics and tests of association with the dependent variable.

Parameters	Mean ± SD (range)/percent (count)	*p*-value
Coach beliefs scale	3.25 ± 0.42 (1.13–4)	
Sex		0.7922ᵃ
Male	36 (38)	
Female	63 (66)	
Country		<0.0001ᵇ
Bosnia	30 (31)	
Lithuania	9 (9)	
Montenegro	19 (19)	
Romania	17 (17)	
Slovakia	28 (29)	
Previous experience		0.0013ᵃ
No	48 (50)	
Yes	53 (55)	
Age		0.5594ᵃ
18–39	56 (58)	
40+	45 (47)	
Familial relationship with athlete		0.0003ᵃ
No	48 (50)	
Yes	53 (55)	
Athlete improvement	4.26 ± 0.58 (2.31–5)	<0.0001ᶜ
Partner improvement	4.48 ± 0.63 (2.62–5)	<0.0001ᶜ
Barriers	2.59 ± 0.9 (1–4)	0.0090ᶜ

Values represent means and standard deviations or percentages. *p* values are based on *t*-tests (ᵃ), one-way ANOVA (ᵇ), or Pearson correlations (ᶜ), depending on variable type.

### 2.2 Procedures

The study was conducted as part of a multi-national evaluation of Unified Sports programs across several European countries. The methodological framework included quantitative survey-based research, using a standardized coach questionnaire to assess relevant psychological, demographic, and attitudinal variables.

Coaches were recruited through Special Olympics organizations and affiliated institutions in each participating country. Local research coordinators facilitated data collection, ensuring adherence to ethical guidelines and cultural sensitivity in the administration of the surveys. Participation was entirely voluntary, and written informed consent was obtained from all participants prior to completing the questionnaire.

As the study involved adult participants and relied solely on anonymous, non-invasive, and non-sensitive questionnaire data, formal ethics committee approval was not required under the national research regulations of the participating countries. No identifiable personal data were collected, and participants were informed of their right to withdraw from the study at any point without any consequences. Nevertheless, all research procedures conformed to internationally accepted ethical standards for research involving human subjects, including the principles outlined in the Declaration of Helsinki. The study protocol was reviewed by the coordinating institution to ensure compliance with these ethical principles.

To ensure linguistic and cultural appropriateness, a rigorous translation and adaptation process was undertaken. The original questionnaire was developed in English and then translated into Romanian, Slovak, Bosnian, Montenegrin, and Lithuanian following a standardized forward-backward translation procedure. Initially, professional translators or bilingual researchers translated the questionnaire into the target languages. These versions were then independently back-translated into English by a separate set of translators unfamiliar with the original English wording. Discrepancies between the original and back-translated versions were reviewed and resolved by the research team to maintain semantic equivalence. Additionally, local coordinators in each country reviewed and evaluated the translated versions for clarity and cultural relevance, and pilot testing was conducted in each country with a small sample of coaches to identify potential misunderstandings or culturally inappropriate terms, leading to minor refinements where necessary.

Data collection was conducted using structured self-completion questionnaires, which were distributed electronically and in paper format during training sessions, workshops, and sports events organized within the Unified Sports framework. Researchers and trained assistants were available on-site to provide clarification if needed. The survey was designed to be accessible and easy to complete, requiring approximately 15–20 min.

### 2.3 Measures

#### 2.3.1 Coach beliefs scale

The dependent variable in this study was coaching attitudes toward Unified Sports, assessed using an adapted version of the Swimming Coaches’ Attitudes Toward Inclusion – Intellectual Disability scale (Hammond et al., 2014). This Coach Beliefs Scale consists of 16 statements assessing the extent to which coaches hold positive or negative attitudes toward coaching athletes with developmental disabilities in Unified Sports programs. The questionnaire consisted of multiple items assessing coaches’ agreement with both positive and skeptical statements regarding inclusion. Some items were reverse-scored to control for response bias, ensuring that higher scores reflected a more positive attitude toward inclusion. Participants rated their agreement with each statement on a 5-point Likert scale (1 = strongly disagree, 5 = strongly agree). The final score for each participant was calculated as the mean of all item responses. A higher score indicated a more favorable attitude toward coaching in Unified Sports, while lower scores reflected more skeptical or negative attitudes toward inclusion. The internal consistency of the Coach Beliefs Scale in this study was high (Cronbach’s α = 0.87).

#### 2.3.2 Perceived athlete improvement

Perceived athlete improvement was measured using two distinct instruments: the Athlete Improvement Scale (AIS) and the Partner Improvement Scale (PIS). These item sets were developed to assess coaches’ perceptions of developmental progress among athletes with developmental disabilities and their non-disabled peers (partners) involved in Unified Sports programs. Respondents rated each item on a 5-point Likert scale ranging from 1 (decrease) to 5 (a lot of improvement), with higher scores reflecting stronger perceptions of improvement.

The AIS consisted of 13 items that captured coaches’ perceptions of progress among athletes with developmental disabilities across a broad range of domains. These included motor abilities (e.g., walking, running, jumping, ball handling, coordination, and balance), social skills (e.g., interaction with other children and adults), communication abilities (e.g., listening and speaking), cognitive functioning (e.g., recognition of body parts, objects, and colors; directional concepts; attention span; memory), adaptive behavior and daily living skills (e.g., following instructions, eye contact, managing materials, putting on a jacket), as well as emotional and social aspects such as self-confidence, self-esteem, emotional expression, decision-making, peer acceptance, and overall well-being. The AIS demonstrated excellent internal consistency in this study (Cronbach’s α = 0.93).

The PIS mirrored this structure with 13 items tailored to measure perceived developmental changes among partners without disabilities. These items included progress in motor and social abilities, communication, cognitive functioning (attention span, memory), understanding and attitudes toward developmental disabilities, self-confidence, decision-making capacity, general well-being and happiness, level of engagement, active participation, self-efficacy, and independence. The PIS also showed high internal consistency (Cronbach’s α = 0.91).

Sample items from the AIS and PIS included prompts such as: “*In general, how would you describe the overall improvement of the athletes in each of the following areas?*” or “*In general, how would you describe the overall improvement of the partners in each of the following areas?*” followed by specific developmental domains such as motor abilities, communication, social interaction, or self-confidence.

The development of both the AIS and PIS was informed by prior studies on inclusive sport participation and developmental outcomes among youth with and without disabilities (e.g., Baran et al., 2009; Hassan et al., 2012; Bota et al., 2014; McConkey et al., 2013), and both instruments were specifically tailored to reflect the goals and structure of Unified Sports programs from the perspective of participating coaches.

#### 2.3.3 Perceived barriers to the implementation of the unified sports program

Perceived barriers were assessed using a set of items asking coaches to evaluate the significance of various obstacles based on their own experience. The instructions stated: “*According to your experience, what were the most significant barriers to the implementation of the Unified Sports program and its goals?*” Each item represented a potential barrier, and respondents rated the degree to which each factor hindered effective implementation on a 5-point Likert scale ranging from 1 (*not a barrier at all*) to 5 (*very significant barrier*), with higher scores reflecting stronger perceived obstacles.

The 23 items covered multiple domains. Organizational and structural barriers included limited program funding, insufficient staff, lack of coach training, and inadequate competitive opportunities. Logistical barriers involved time constraints, transportation difficulties, lack of sports equipment, and insufficient access to facilities. Social and cultural barriers reflected negative societal attitudes, limited parental support, or resistance from other coaches and administrators. Athlete-specific barriers addressed concerns such as behavioral challenges, communication difficulties, or limitations in athletes’ physical and cognitive abilities. This barrier scale demonstrated good internal consistency (Cronbach’s α = 0.89).

This multidimensional tool was based on previous research on inclusion-related barriers in sport settings (e.g., Jaarsma et al., 2014; Ballas et al., 2022; Grandisson et al., 2021) and was tailored to capture Unified Sports-specific implementation challenges from the coach’s perspective.

#### 2.3.4 Demographic and professional characteristics

To explore additional factors influencing coaching attitudes, demographic and professional background information was collected. Gender was recorded as male or female. Age was reported as a continuous variable. Educational background was categorized into high school education and university degree. Coaching experience was measured in terms of years of experience working with athletes with and without developmental disabilities. Employment setting was identified as school-based, Special Olympics-affiliated, or other coaching environments. Prior exposure to Unified Sports was assessed by asking coaches how many years they had been involved in coaching Unified Sports teams. Personal connection to athletes was determined by asking whether the coach had a familial relationship with an athlete in the program.

These variables were examined as correlates of coaching attitudes toward Unified Sports to provide a broader understanding of the factors shaping coaches’ perspectives and experiences in inclusive sports programs.

### 2.4 Statistical analysis

All statistical analyses were conducted using TIBCO Software Inc. (2017). Statistica (data analysis software system), version 13. Descriptive statistics were used, including mean values, standard deviations (SD), minimum and maximum ranges, frequencies, and percentages. To assess the strength and significance of relationships between independent variables and the dependent variable (coaches’ beliefs), *t*-tests, one-way analysis of variance (ANOVA), and Pearson’s correlation coefficients were applied, depending on the type of variable (Table 1). A hierarchical multiple regression analysis was conducted, with coaches’ beliefs as the dependent variable. Only those variables that showed statistically significant associations with belief levels and met other inclusion criteria were entered into the models. The level of statistical significance was set at *p* < 0.05.

## 3 Results

### 3.1 Preliminary analyses and descriptive results

Descriptive statistics and inferential analyses were conducted to examine the associations between demographic, experiential, and psychological variables and coaches’ attitudes toward Unified Sports. No significant differences were observed between male and female coaches (*p* = 0.7922), nor across age groups (*p* > 0.05), suggesting that gender and age were not influential factors in shaping coaching attitudes within this sample (see Table 1). However, statistically significant differences were found between countries (*p* < 0.0001), indicating variability in average belief scores across national groups (Table 1). While specific pairwise comparisons were not conducted, this result justified controlling for country in subsequent models or acknowledging national context in interpretation.

A significant effect was also observed for previous coaching experience with athletes with disabilities (*p* = 0.0013), with experienced coaches tending to score differently on the beliefs scale (Table 1) than those without such experience. Similarly, coaches who reported a familial relationship with an athlete (e.g., parent or sibling) showed significantly different attitudes compared to those without such a connection (*p* = 0.0003) (Table 1). These findings suggest that both personal involvement and experiential background may be associated with variation in coaching attitudes. Furthermore, continuous variables representing perceived improvement among athletes and partners were positively associated with coaching attitudes (*p* < 0.0001 for both), indicating that coaches who observed more developmental progress tended to report more favorable beliefs toward Unified Sports (Table 1). Although both scales were positively correlated with beliefs, only athlete improvement emerged as a predictor in the regression model, as discussed below. Perceived barriers also showed a modest but statistically significant correlation with coaching beliefs (*p* = 0.009) (Table 1), though this relationship did not remain significant in the multivariate analysis. These preliminary findings provided the empirical basis for variable selection in the hierarchical regression analysis.

### 3.2 Hierarchical multiple regression

A hierarchical multiple regression analysis was conducted, with coaching attitudes as the dependent variable. Variables included in the models were those that demonstrated statistically significant associations with coaching attitudes in preliminary analyses and met theoretical or methodological criteria for inclusion (Table 1). The models were statistically significant (*p* < 0.05) and explained up to 32% of the variance (Table 2).

**Table 2:** Hierarchical regression analysis predicting coaches beliefs.

Step	Predictor	b*	*R* ^2^	Δ*R*^2^	*F*	*p*
Step1			0.17		20.67	<0.0001
	Athlete improvement	0.41				<0.0001
Step 2			0.28	0.11	20.21	<0.0001
	Athlete improvement	0.4				<0.0001
	Familial relationship with athlete	−0.34				0.0001
Step 3			0.32	0.04	16.12	<0.0001
	Athlete improvement	0.4				<0.0001
	Familial relationship with athlete	−0.27				0.0029
	Previous experience	−0.21				0.0163

The level of coaches’ beliefs (dependent variable) was predicted based on three predictors entered sequentially: perceived athlete improvement, familial relationship with athlete, and previous experience. In the first step, perceived athlete improvement was entered into the model, explaining 17% of the variance. In the second step, familial relationship with athlete was added, increasing the explained variance by an additional 11%. In the third step, previous experience was included. All three variables were significant predictors of coaches’ beliefs (see Table 2). Perceived barriers were not a statistically significant predictor in the final regression model, indicating that their contribution to the explained variance in coaching attitudes was not incremental beyond other included variables.

## 4 Discussion

This multi-country study examined how perceived athlete improvement, prior coaching experience, and selected contextual factors were related to coaches’ attitudes toward Unified Sports. Of the variables examined, perceived improvement in athletes with developmental disabilities emerged as the strongest correlate of positive coaching attitudes. This finding is consistent with previous research indicating that observable progress in athletes is an important motivator for coaches, increasing their commitment to inclusive sport environments (Mageau and Vallerand, 2003; Moen and Federici, 2013; Sakalidis et al., 2023). Specifically, when coaches perceive that athletes are making tangible gains in skills, social interaction, or confidence, they are more likely to adopt supportive and sustainable attitudes toward Unified Sports programs.

Interestingly, perceived improvement in non-disabled partners—although rated positively overall—did not contribute significantly to the prediction of coaching attitudes in this study. This suggests that coaches may prioritize developmental gains observed in athletes with disabilities over those observed in their non-disabled peers when forming their perspectives on Unified Sports. A possible explanation for this is that improvements in athletes with developmental disabilities are more salient, particularly in relation to the primary inclusion goals of the program. In addition, coaches may perceive the progress of athletes with disabilities as a more direct indicator of the program’s impact, and thus more closely linked to their own sense of efficacy and motivation. While previous studies have emphasized that Unified Sports fosters mutual benefits—including increased social understanding and collaboration between athletes with and without disabilities (Bota et al., 2014; Pan and Davis, 2019)—our findings highlight a potential asymmetry in how these benefits are weighted by coaches. Although partners may benefit in areas such as empathy, teamwork and inclusive attitudes, these perceived changes appear to play a less central role in shaping coaches’ overall attitudes toward inclusive coaching environments.

The presence of a familial relationship with an athlete was also a significant predictor of coaching attitudes. Coaches who reported such ties expressed more negative beliefs overall, which may reflect emotional over-involvement, protective tendencies, or challenges in role separation between family and coaching responsibilities. While this finding might initially appear counterintuitive, a recent systematic review by McShan and Moore (2023) highlights that emotionally complex or family-based coach–athlete relationships may introduce additional interpersonal stressors. These can complicate role clarity and increase emotional burden, particularly in inclusive environments, which may in turn influence attitudes toward Unified Sports. This area warrants further exploration, especially in programs where volunteer or family-involved coaching is common.

Contrary to some prior studies (Jaarsma et al., 2014; Ballas et al., 2022), perceived barriers were not a significant predictor of coaching attitudes when controlling for other variables. Although many coaches identified institutional, logistical, and social barriers to inclusion, these barriers did not appear to independently predict their beliefs about Unified Sports. One explanation may be that coaches who observe meaningful progress in their athletes are more resilient to such barriers or perceive them as manageable challenges. This is consistent with previous case study research suggesting that perceived success and positive athlete outcomes can help coaches manage or reinterpret systemic barriers, leading to sustained engagement despite challenges (Matsunaga, 2019).

Unexpectedly, previous experience working with athletes with developmental disabilities was associated with more negative attitudes toward Unified Sports. While earlier studies have generally linked experience to more positive or inclusive coaching perspectives (e.g., Hassan and Lynch, 2014; Mauro et al., 2021), our findings suggest that the relationship may be more complex. One possible explanation is that coaches with longer or more sustained involvement in Unified Sports may face different challenges that were not fully captured in this study—such as emotional fatigue, perceived lack of progress, or insufficient systemic support—which could influence their attitudes over time. It is also conceivable that experienced coaches develop higher expectations regarding program structure or effectiveness, which, when unmet, may result in greater skepticism. While speculative, these possibilities highlight the need for future research to explore not only the quantity of experience but also its quality, including access to training, institutional support, and long-term outcomes. Understanding these contextual nuances may help explain why, in some cases, experience does not necessarily translate into more favorable attitudes.

The complexity of these relationships reflects the diverse challenges coaches face in implementing Unified Sports programs. As the literature suggests, attitudes are shaped not only by individual characteristics or isolated perceptions, but also by a broader interaction between perceived progress, emotional investment, and contextual enablers or constraints (McConkey et al., 2021; Hammond et al., 2014). This points to the importance of designing coach development initiatives that address both cognitive and emotional dimensions of inclusive coaching—by providing practical tools, creating opportunities for reflection, and fostering a supportive peer network.

While the study contributes valuable insights, several limitations should be acknowledged. First, the cross-sectional design limits the ability to infer causality between the investigated variables. Second, although the study included data from five countries, the unequal group sizes prevented meaningful between-country comparisons and the overall sample size limits the generalizability of findings to broader coaching populations. Future studies should explore cross-national differences in more balanced samples or using multilevel modeling techniques. Third, the reliance on self-report data may introduce social desirability bias, particularly when assessing sensitive topics such as attitudes or personal connections. Lastly, while the perception of athlete improvement emerged as a key correlate, the study did not measure actual athlete outcomes, which could provide important triangulation in future evaluations. Despite these limitations, the inclusion of coaches from multiple national contexts enhances the ecological validity of the findings and highlights shared patterns that may be relevant across diverse implementation environments. Nevertheless, caution is warranted when interpreting the findings beyond the specific sample examined.

Future research should further explore the psychological mechanisms through which perceptions of improvement influence coaching motivation, possibly incorporating constructs such as coach self-efficacy, perceived competence, or emotional reward. Additionally, qualitative studies may provide richer insight into how familial relationships or prior experiences are experienced by coaches, especially in resource-constrained environments.

## 5 Conclusion

This study identified perceived improvement among athletes with developmental disabilities as a central correlate of positive coaching attitudes toward Unified Sports, highlighting the importance of visible progress in sustaining coach motivation and commitment. Contrary to expectations, prior coaching experience and familial ties with athletes were associated with less favorable attitudes, suggesting that emotional or contextual complexities may influence perceptions in nuanced ways. Although perceived barriers were frequently acknowledged, they did not independently predict coaching beliefs when other variables were accounted for. These findings underscore the need for targeted support structures that address not only logistical challenges but also the emotional and experiential dimensions of inclusive coaching. Further research should explore how different coaching environments and support systems shape long-term engagement in Unified Sports.

## Data Availability

The raw data supporting the conclusions of this article will be made available by the authors, without undue reservation.

## References

[ref1] Ballas J. Buultjens M. Murphy G. Jackson M. (2022). Elite-level athletes with physical impairments: barriers and facilitators to sport participation. Disabil. Soc. 37, 1018–1037. doi: 10.1080/09687599.2020.1862642

[ref2] Baran F. Top E. Aktop A. Özer D. Nalbant S. (2009). Evaluation of a unified football program by Special Olympics athletes, partners, parents, and coaches. Eur. J. Adapt. Phys. Activity 2, 34–45. doi: 10.5507/euj.2009.003, PMID: 27504511

[ref3] Bota A. Teodorescu S. Şerbănoiu S. (2014). Unified sports – a social inclusion factor in school communities for young people with intellectual disabilities. Procedia. Soc. Behav. Sci. 117, 21–26. doi: 10.1016/j.sbspro.2014.02.172

[ref4] Conatser P. Block M. Gansneder B. (2002). Aquatic instructors’ beliefs toward inclusion: the theory of planned behavior. Adapt. Phys. Act. Q. 19, 172–187. doi: 10.1123/apaq.19.2.172, PMID: 28195776

[ref5] Dowling S. (2014). “Sport and intellectual disability. Benefits, barriers and bridges” in Sport, coaching and intellectual disability. eds. Hassan D. Dowling S. McConkey R. (London New York: Routledge), 34–51.

[ref6] Grandisson M. Chrétien-Vincent M. Ouellet B. Marcotte J. Lamontagne M. E. Milot É. (2021). Survey on strategies to promote social inclusion through sports. Inc 9, 104–117. doi: 10.1352/2326-6988-9.2.104

[ref7] Hammond A. M. (2022). The relationship between disability and inclusion policy and sports coaches’ perceptions of practice. Int. J. Sport Policy Politics 14, 471–487. doi: 10.1080/19406940.2022.2074515

[ref8] Hammond A. M. Jeanes R. Penney D. Leahy D. (2019). “I feel we are inclusive enough”: examining swimming coaches’ understandings of inclusion and disability. Sociol. Sport J. 36, 311–321. doi: 10.1123/ssj.2018-0164

[ref9] Hammond A. M. Young J. A. Konjarski L. (2014). Attitudes of Australian swimming coaches towards inclusion of swimmers with an intellectual disability: an exploratory analysis. Int. J. Sports Sci. Coach. 9, 1425–1436. doi: 10.1260/1747-9541.9.6.1425

[ref10] Hassan D. Dowling S. McConkey R. Menke S. (2012). The inclusion of people with intellectual disabilities in team sports: lessons from the youth unified sports programme of Special Olympics. Sport Soc. 15, 1275–1290. doi: 10.1080/17430437.2012.695348

[ref11] Hassan D. Lynch R. (2014). “Reflections on coaching athletes with disabilities” in Sport, coaching and intellectual disability. eds. Hassan D. Dowling S. McConkey R. (London New York: Routledge), 69–84.

[ref12] Jaarsma E. A. Dijkstra P. U. Geertzen J. H. B. Dekker R. (2014). Barriers to and facilitators of sports participation for people with physical disabilities: a systematic review. Scand. J. Med. Sci. Sports 24, 871–881. doi: 10.1111/sms.12218, PMID: 24730752

[ref13] Kandianos E. Fadeeva A. Hettinga F. J. Ling F. C. M. (2023). The role of the social environment in inclusive sports participation—identifying similarities and challenges in athletes with and without intellectual disabilities through coaches’ eyes: a qualitative inquiry. PLoS One 18:e0280379. doi: 10.1371/journal.pone.0280379, PMID: 36630463 PMC9833589

[ref14] Khetani M. A. Graham J. E. Davies P. L. Law M. C. Simeonsson R. J. (2015). Psychometric properties of the young children's participation and environment measure. Arch. Phys. Med. Rehabil. 96, 307–316. doi: 10.1016/j.apmr.2014.09.031, PMID: 25449189 PMC4306635

[ref15] MacDonald D. J. Beck K. Erickson K. Côté J. (2016). Understanding sources of knowledge for coaches of athletes with intellectual disabilities. J. Appl. Res. Intellect. Disabil. 29, 242–249. doi: 10.1111/jar.12174, PMID: 25846939

[ref16] Mageau G. A. Vallerand R. J. (2003). The coach–athlete relationship: a motivational model. J. Sports Sci. 21, 883–904. doi: 10.1080/0264041031000140374, PMID: 14626368

[ref17] Matsunaga K. (2019). Inclusion through sport: A case study of coaches' experiences of Special Olympics unified sports (doctoral dissertation). ProQuest Dissertations & Theses.

[ref18] Mauro A. Bruland D. Latteck Ä.-D. (2021). “With enthusiasm and energy throughout the day”: promoting a physically active lifestyle in people with intellectual disability by using a participatory approach. Int. J. Environ. Res. Public Health 18:12329. doi: 10.3390/ijerph182312329, PMID: 34886054 PMC8656824

[ref19] McConkey R. Dowling S. Hassan D. Menke S. (2013). Promoting social inclusion through unified sports for youth with intellectual disabilities: a five-nation study. J. Intellect. Disabil. Res. 57, 923–935. doi: 10.1111/j.1365-2788.2012.01587.x, PMID: 22672339

[ref20] McConkey R. Pochstein F. Carlin L. Menke S. (2021). Promoting the social inclusion of players with intellectual disabilities: an assessment tool for sport coaches. Sport Soc. 24, 430–439. doi: 10.1080/17430437.2019.1673369

[ref21] McShan K. Moore E. W. G. (2023). Systematic review of the coach–athlete relationship from the coaches’ perspective. Kinesiol. Rev. 12, 158–173. doi: 10.1123/kr.2022-0006

[ref22] Moen F. Federici R. A. (2013). Coaches’ coaching competence in relation to athletes’ perceived progress in elite sport. J. Educ. Learn. 2, 240–252. doi: 10.5539/jel.v2n1p240

[ref23] Orbán-Sebestyén K. Szilárd Z. S. Farkas J. Ökrös C. Roswal G. M. (2023). Attitude of elite tennis coaches working with athletes with intellectual disabilities participating in Special Olympics. J. Intellect. Disabil. Res. 67, 123–135. doi: 10.1111/jir.12996, PMID: 36437706

[ref24] Pan C. C. Davis R. (2019). Exploring physical self-concept perceptions in athletes with intellectual disabilities: the participation of unified sports experiences. Int. J. Dev. Disabil. 65, 293–301. doi: 10.1080/20473869.2018.1470787, PMID: 34141350 PMC8115608

[ref25] Rizzo T. L. (1984). Attitudes of physical educators toward teaching handicapped pupils. Adapt. Phys. Act. Q. 1, 267–274. doi: 10.1123/apaq.1.4.267

[ref26] Rizzo T. L. Bishop P. Tobar D. (1997). Attitudes of soccer coaches toward youth players with mild mental retardation: a pilot study. Adapt. Phys. Act. Q. 14, 238–251. doi: 10.1123/apaq.14.3.238

[ref27] Sakalidis K. E. Hettinga F. J. Ling F. C. M. (2023). Coaching styles and sports motivation in athletes with and without intellectual impairments. PLoS One 18:e0296164. doi: 10.1371/journal.pone.0296164, PMID: 38134184 PMC10745216

[ref28] Svanelöv E. Wallén E. F. Enarsson P. Stier J. (2020). ‘Everybody with disability should be included’: a qualitative interview study of athletes’ experiences of disability sports participation analysed with ideas of able-mindedness. Scand. J. Disabil. Res. 22, 296–306. doi: 10.16993/sjdr.676

[ref29] Temple V. A. Walkley J. W. (1999). Academic learning time—physical education (ALT-PE) of students with mild intellectual disabilities in regular Victorian schools. Adapt. Phys. Act. Q. 16, 64–74. doi: 10.1123/apaq.16.1.64

[ref30] Tsai E. H. Fung L. (2009). Parents’ experiences and decisions on inclusive sport participation of their children with intellectual disabilities. Adapt. Phys. Act. Q. 26, 151–171. doi: 10.1123/apaq.26.2.151, PMID: 19478347

[ref31] Vargas T. M. Flores M. M. Beyer R. (2012). Coaching athletes with hidden disabilities: recommendations and strategies for coaching education. Strateg.: J. Phys. Sport Educ. 25, 32–33. doi: 10.1080/08924562.2012.10592150

[ref32] Wilski M. Nadolska A. Dowling S. McConkey R. Hassan D. (2012). Personal development of participants in Special Olympics unified sports teams. Hum. Movem. 13, 271–279. doi: 10.2478/v10038-012-0032-3, PMID: 40529641

